# Immunolocalization and proteomic analyses of IZUMO1 in porcine spermatozoa

**DOI:** 10.3389/fcell.2025.1576881

**Published:** 2025-05-16

**Authors:** Miranda Hernández-Falcó, Paula Sáez-Espinosa, Andrea López-Botella, Laura Robles-Gómez, Francisco Alberto García-Vázquez, Maria José Izquierdo-Rico, Pedro José Llamas-López, María José Gómez-Torres

**Affiliations:** ^1^ Department of Biotechnology, University of Alicante, Alicante, Spain; ^2^ Departamento de Fisiología, Facultad de Veterinaria, Universidad de Murcia, Murcia, Spain; ^3^ Instituto Murciano de Investigación Biosanitaria (IMIB-Pascual Parrilla), Murcia, Spain; ^4^ Departamento de Biología Celular e Histología, Facultad de Medicina, Universidad de Murcia, Murcia, Spain; ^5^ Department of Agri-Food Technology, University of Miguel Hernández, Elche, Spain; ^6^ Human Fertility Cathedra, University of Alicante, Alicante, Spain

**Keywords:** acrosome reaction, boar, capacitation, immunofluorescence, IZUMO1, proteomics

## Abstract

Reproduction is fundamental to breeding programs aimed at increasing productivity in swine industry. However, the application of *in vitro* embryo production in this species is limited because of the polyspermy. Therefore, characterizing proteins involved in sperm-oocyte binding such as IZUMO1 becomes essential. This study aimed to characterize porcine IZUMO1 protein under three different physiological states: sperm-rich fraction (SRF), 1-h capacitated sperm selected by *swim-up* (CS), and induced acrosome reaction in 1-h capacitated sperm (ARS). The immunolocalization of IZUMO1 and acrosome status of fifteen fertile boars was assessed by confocal microscopy. Additionally, six males were subjected to a more detailed examination via quantitative proteomic analysis by LC–MS/MS. Fluorescence results revealed four distinct IZUMO1 distribution patterns: pattern 1 (P1) characterized by speckled staining in the pre-equatorial subdomain and postacrosomal domain, pattern 2 (P2) displaying strong apical ridge staining with speckled staining in the pre-equatorial subdomain and postacrosomal domain, pattern 3 (P3) exhibiting speckled staining in the postacrosomal domain, and pattern 4 (P4) without labelling. In the SRF sperm, IZUMO1 was predominantly distributed between staining patterns P1 and P2 (∼50%). As a result of the capacitation, there was a significant decrease in P1. Conversely, in ARS, IZUMO1 was dominantly distributed in P3 51.55% and P4 24.25%. The quantitative study of the IZUMO1 protein supported these findings. With those results and compared with our previous work in human, here we propose a working model of IZUMO1 migration dependent on the morphology and subdomains of the sperm head.

## 1 Introduction

Spermatozoa, propelled by chemotactic signals and capacitation, navigate the female reproductive tract to reach the oocyte (De Jonge, 2017). The encounter between sperm and oocyte initiates a series of events, commencing with the recognition and binding of sperm to the zona pellucida (ZP). This interaction triggers the acrosome reaction, during which enzymes are released to facilitate sperm penetration through the ZP (Breitbart and Grinshtein, 2023). It should be noted that a variable percentage of spermatozoa may undergo spontaneous acrosomal reaction (La Spina et al., 2016). Upon successful penetration, the fusion of the gamete membranes occurs, leading to the formation of a zygote. Simultaneously, this fusion prompts a cascade of intracellular signaling events, including the release of intracellular calcium ions, which culminate in the prevention of polyspermy (Bianchi and Wright, 2020). The intricate mechanism of mammalian fertilization involves precise molecular and cellular events, and any deviations from this orchestrated process can lead to reproductive challenges.

Molecular players, such as the IZUMO1 protein, serve as indispensable orchestrators, facilitating the fusion of gametes and the deposition of genetic material (Brukman et al., 2023). Understanding the molecular dynamics of fertilization in porcine species not only enhances our knowledge of species-specific reproductive biology, but also contributes to the advancement of assisted reproduction technologies and selective breeding techniques. The impact of fertilization in swine extends beyond reproduction, affecting on industry structure, production, efficiency, quality, and profitability.

The essential protein IZUMO1 is composed of two domains. Specifically, the IZUMO domain consists of four α-helices (α1, α2, α3, α4) and two antiparallel β-strands (β1, β2). Brukman et al. (2023), have shown the existence of three aromatic residues in α4 (F28A, W88A, W113A) that mediate fusion independently of IZUMO1-JUNO interaction. Interestingly, an adhesion-related residue was reported for the first time in the β-hairpin. In this way, IZUMO1 plays a role in both adhesion and fusion of gametes. Additionally, the immunoglobulin-like domain would not be involved in fusion, but rather in the movement and localization of the protein.

Hence, the primary objective of this study was to characterize IZUMO1 in pig spermatozoa. Due to the changes that the spermatozoa undergo during the female reproductive tract spermatozoa were studied under different physiological states: sperm-rich fraction (SRF), 1-h capacitated sperm selected by *swim-up* (CS), and induced acrosome reaction in 1-h capacitated sperm (ARS). For this purpose, the distribution of IZUMO1 in the sperm head was analyzed in accordance with the acrosomal status. Proteomic studies were also carried out to compare the expression of IZUMO1 in the different physiological states.

## 2 Material and methods

### 2.1 Sample preparation

A total of 15 ejaculated (sperm-rich fraction) from different fertility-tested Pietrain boars (Axiom Genetics line) were purchased from a swine specialized artificial insemination center (SPERMATICA REPRODUCCION SL., Lorca, Spain). Sperm concentration, motility, and morpho-anomalies were evaluated using a commercial computer-assisted sperm analysis system (CASA system, Microptic, Barcelona, Spain) meanwhile viability was corroborated with VitalStain™ (NidaCon International AB, Mölndal, Sweden). The inclusion criteria established for using the ejaculates were: total motility >70%, viable sperm >90%, and morpho-anomalies ≤30%.

### 2.2 Sperm capacitation by *swim-up*

The *swim-up* selection (Boiti et al., 2005) consisted in transfer of 100 μL of the sample to the bottom of an Eppendorf tube containing 1 mL of TALP capacitation medium (Robles-Gómez et al., 2021). Finally, the supernatant fraction (900 μL) was collected and washed three times with sterile-filtered Dulbecco´s phosphate buffered saline (PBS, Capricorn Scientific GmbH, Ebsdorfergrund, Germany) by centrifugation (200 g, 5 min).

### 2.3 Induction and evaluation of acrosomal status

Acrosome reaction was induced in the recovered spermatozoa by adding 10 μM of calcium ionophore A23187 (Sigma-Aldrich®, Saint Louis, MO, United States) and 2 mM of calcium chloride (Panreac Química S.L.U, Barcelona, Spain) at 38.5°C with 5% (v/v) CO_2_ for 1 h following previous protocols of our group (Robles-Gómez et al., 2021).

Sperm acrosome status was assessed in methanol-fixed samples with *Peanut agglutinin* lectin conjugated with fluorescein-5-isothiocyanate (PNA-FITC, Vector Laboratories Burlingame, CA) at a final concentration of 3 mg/mL for 30 min. Samples were mounted using Fluoroshield™ with 4′,6-diamidine-2′-phenylindole dihydrochloride (Sigma-Aldrich®, Saint Louis, MO, United States).

### 2.4 Fixation

All sperm physiological states were divided into two aliquots. One of them was fixed in 2% (w/v) paraformaldehyde (Electron MicroscopySciences, Hatfield, PA, United States) and stored at 4°C until their use. The other aliquot was stored unfixed at −80°C for proteomic analysis.

### 2.5 Western blot

SRF was mixed with an equal volume of 4X SDS sample buffer (Merck Millipore Ltd., County Cork, Ireland) and heated in a Thermo Mixer for 10 min at 95°C. After removing sperm debris proteins were separated by SDS-PAGE (NovexTM WedgeWellTM 16% Tris–Glycine Gel, Invitrogen, Waltham, MA, United States) and transferred to Immobilon-P PVDF membrane (Merck Millipore Ltd., County Cork, Ireland) at 40 V for 75 min. The membrane was subsequently blocked in TBST-1%BSA and incubated with rabbit anti-IZUMO1 polyclonal antibody (ref. orb184402, Biorbyt Ltd., Cambridge, United Kingdom) at 1:1000 in TBST-1%BSA and mouse anti-rabbit IgG-HRP (sc-2357, Santa Cruz Biotechnology, Germany) at 1:10,000 in TBST-1%BSA. After washing, the membrane was incubated with 1 mL Pierce® ECL Plus Western blotting substrate (Thermo Fisher Scientifc, Roskilde, Denmark) at RT for 3 min. Chemiluminescence signal was acquired by ImageQuant LAS500 (GE Healthcare Bio- Sciences AB, Uppsala, Sweden).

### 2.6 IZUMO1 immunostaining

Immunolabeling of IZUMO1 was performed following the methodology previously described by our group (Gómez-Torres et al., 2023). Smears were permeabilized with 0.5% (w/v) Triton X-100 and blocked in 2% (w/v) BSA-PBS. Afterward, smears were incubated with anti-IZUMO1 antibody (1:100) overnight at 4°C and 1 h at RT in darkness with a secondary anti-rabbit antibody conjugated with Cy^3^ (1:100, Jackson ImunoResearch, Ely, United Kingdom). For the co-staining of IZUMO1 and acrosome status, samples were additionally incubated with PNA-FITC during 30 min. Finally, the smears were washed and mounted using Fluoroshield™ with DAPI.

### 2.7 LC-MS/MS analysis

The proteomic analysis was performed in the proteomics facility of SCSIE University of Valencia. This proteomics laboratory is a member of Proteored. In-solution samples of six randomly selected males under all physiological states (SRF, CS, and ARS) and in-gel samples from SRF were used.

Total protein from in-solution extracts were quantified using Machery Nagel kit (Machery-Nagel, Dueren, Germany). Specifically, 5 μg total protein extracts of each sample were dissolved in 20 μL of 50 mM ammonium bicarbonate and processed according SP3 protocol. Then, 7 μL of beads concentrated at 20 μg/μL were added to each sample and then, immediately, acetonitrile (ACN) was added to a final concentration of 70% (v/v). The mixture was briefly vortexed, incubated for 20 min at RT and placed on a magnetic rack for 2 min, discarding the supernatant. The beads were then washed twice with 70% EtOH, and once with CAN. For elution, the washed beads were resuspended in 50 mM AMBIC buffer and treated overnight with trypsin (200 ng, Promega, WI, United States) at 37°C with agitation. Digestions were stopped with 10% trifluoroacetic acid (TFA) (Thermo Fisher Scientific, Fremont, DA, United States). The tubes were centrifuged at 20,000 *g* for 1 min and the supernatants containing peptides were collected.

In the case of in-gel samples, the proteins were stained with PageBlue™ Protein Satining Solution (Thermo Fisher Scientifc, Roskilde, Denmark). The gel slides were fragmented and digested with sequencing grade trypsin (250 ng) (Shevchenko et al., 1996). Digestion was set at 37°C with agitation, from 5 h. The trypsin digestion was stopped with 10% TFA.

The final peptide mixtures from in-solution or in-gel samples were analyzed by liquid chromatography and tandem mass spectrometry (LC–MS/MS) in a TimsTOF fleX mass spectrometer (Bruker, Billerica, MS, United States). First, 5 µL of SP3 digested protein mixture was diluted to 20 μL with 0.1% FA, loaded in an Evotip pure tip (EvoSep, Odense, Denmark), eluted to an analytical column (EvoSep 15 cm × 150 μm, 1.5 μm; Evosep, Odense, Denmark) by the Evosep One system and resolved with the 30 SPD chromatographic method defined by the manufacturer. The eluted peptides were ionized in a captive Spray with 1700 V at 200°C. MSn data were acquired in data independent acquisition mode (DIA) and using Parallel Accumulation Serial Fragmentation (PASEF) with base method m/z range of 100–1700 m/z and 1/k 0 0.6-1.6 Vs/cm^2^ using cycle time of 100 m locked to 100% duty cycle. The system sensitivity was controlled with 50 ng of HELA digested proteins.

In-solution data analysis was performed using PaSER system (Bruker, Billerica, MS, United States) to send the data to the integrated software suite DIA-NN (version 1.8) for identification and quantification of proteins. For this purpose, an in silico-predicted spectral library out of a sequence database (uniprot_*sus scrofa*. fasta) was created by DIA-NN. Then, diaPASEF raw data files were analysed. The quantification strategy was set to QuantUMS (high precision), single pass mode neural network classifier, RT-dependent and optimal result. Output main DIA-NN reports were filtered with a global FDR ≤1% on both unique genes and proteins group levels.

In-gel data analysis was performed using both PaSER system and MSFragger via FragPipe. The presence of IZUMO1 was corroborated by accepting those specific peptides with a confidence ≥90%.

### 2.8 Statistical analysis

A minimum of 200 cells were evaluated in each condition using a Confocal Laser Scanning Zeiss LSM 800 Microscope (Zeiss, Oberkochen, Germany) with an oil 100x objective and 405 nm, 488 nm, and 561 nm lasers. The IZUMO1 staining pattern in the sperm head and acrosome were quantified as percentages (%).

The Shapiro-Wilk test showed that the IZUMO1 location was normally distributed for each sperm physiological status (SRF, CS, and ARS). Two-way analysis of variance (ANOVA), univariate analysis and Bonferroni *post hoc* test were used to assess the significant labeling differences between groups. Descriptive (mean ± standard deviation; SD) and statistical results were obtained using IBM SPSS Statics 28.0 (IBM, Armonk, NY). Two-sided *p* ≤ 0.05 was deemed statistically significant. The area data obtained with DIA-NN was analyzed with Marker View (Sciex, Framingham, MA, United States).

## 3 Results

### 3.1 Assessment of acrosomal status

**FIGURE 1:**
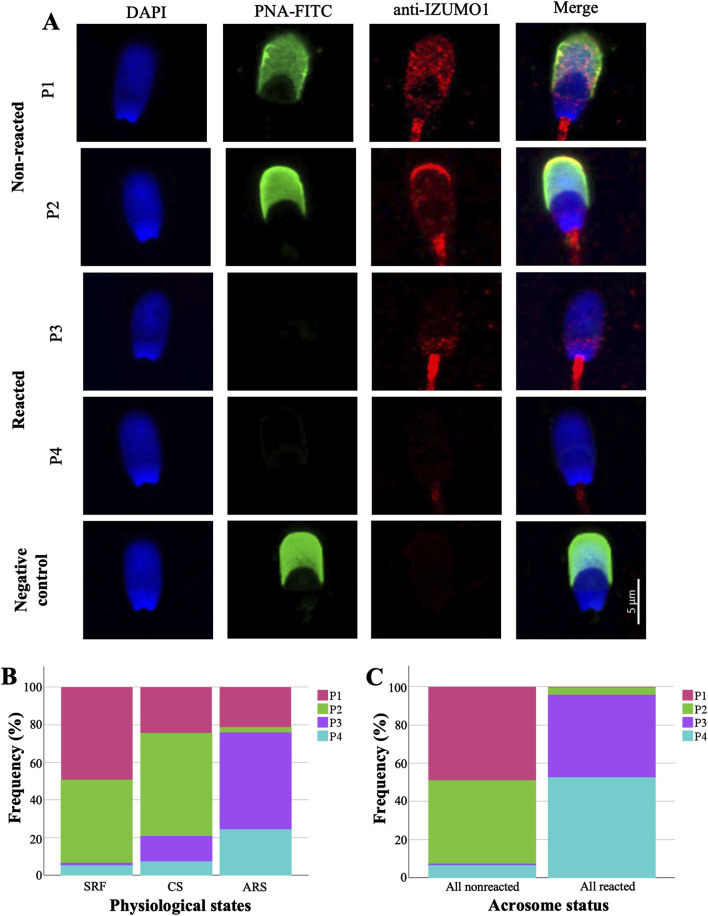
Analysis and distribution of IZUMO1 staining patterns in porcine sperm. **(A)** Representative images of IZUMO1 and acrosome status co-staining. **(B)** Percentages of IZUMO1 staining patterns in each physiological state. **(C)** IZUMO1 frequency staining patterns in all pig spermatozoa according to acrosomal status. Pattern 1 (P1) characterized by speckled staining in the pre-equatorial subdomain and postacrosomal domain, pattern 2 (P2) displaying strong apical ridge staining with speckled staining in the pre-equatorial subdomain and postacrosomal domain, pattern 3 (P3) exhibiting speckled staining in the postacrosomal domain, and pattern 4 (P4) without labelling. Sperm-rich fraction (SRF), 1-h capacitated sperm selected by *swim-up* (CS), induced acrosome reaction in 1-h capacitated sperm (ARS).

Cells showing green fluorescence in the acrosome were classified as non-reacted, while those without labeling, were considered reacted (see Figure 1A). The spontaneous acrosome reaction rate prior to *swim-up* capacitation was 3.46% ± 1.71%, compared to 23.52% ± 10.18% after capacitation process (SRF vs. CS). Moreover, the induced acrosome reaction percentage in ARS was 67.33% ± 14.24%. Significantly different results were observed among the experimental conditions (*p* ≤ 0.001).

### 3.2 Antibody validation

**FIGURE 2:**
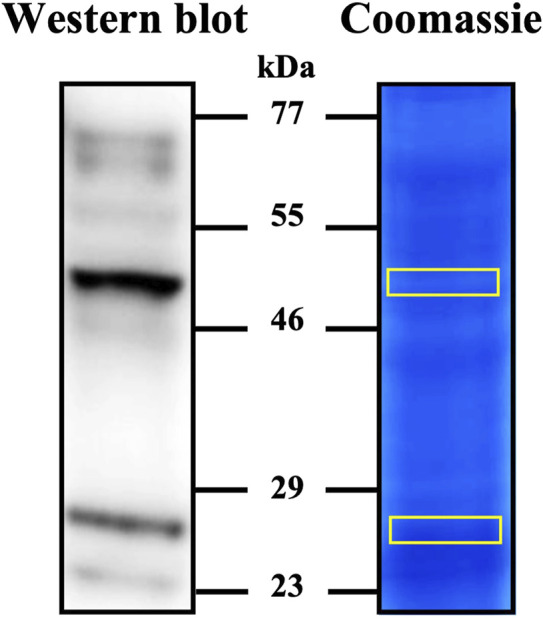
Detection of IZUMO1 in pig spermatozoa. Western blot stained with anti-IZUMO1 antibody and gel stained with colloidal coomassie G-250 (PageBlue™ Protein Satining Solution). After staining, the bands marked in yellow on the coomassie were cut and analyzed by LC–MS/MS to corroborate the presence of IZUMO1.

The specificity of anti-IZUMO1 antibody against porcine was evaluated by Western blotting and in-gel sequencing by LC-MS/MS (Figure 2). Western blot of the extracts from sperm-rich fraction revealed a major form of ∼48-kDa and a minor form of ∼28-kDa. In-gel sequencing identified three peptides with 99% of confidence for IZUMO1.

### 3.3 Immunolocation of IZUMO1 in spermatozoa

To understand the distribution of IZUMO1 it should be noted that the porcine sperm head is divided into domains and subdomains (see Supplementary Datasheet S1). Figure 1A illustrates four distinct labeling patterns observed for IZUMO1: pattern 1 (P1) characterized by speckled staining in the pre-equatorial subdomain and postacrosomal domain, pattern 2 (P2) displaying strong apical ridge staining with speckled staining in the pre-equatorial subdomain and postacrosomal domain, pattern 3 (P3) exhibiting speckled staining in the postacrosomal domain, and pattern 4 (P4) without labelling.

Both non-capacitated and capacitated spermatozoa displayed similar distribution patterns of labeling. Specifically, IZUMO1 was predominantly distributed between staining patterns P1 (SRF 49.31% vs. 24.58% CS) and P2 (∼50%). However, in 13.55% of CS cells, the staining pattern P3 was observed. In contrast, in ARS, IZUMO1 was significantly (*p* ≤ 0.001) and predominantly distributed in the postacrosomal domain (P3 51.55%) or was no longer detectable (P4 24.25%; Figure 1B). A detailed table of the statistical data of the patterns of IZUMO1 staining under different physiological states (SRF, CS, and ARS) can be found in the Supplementary Datasheet S1.

Upon detailed analysis of IZUMO1 localization depending on the acrosomal status via co-labeling, significant differences were observed between total non-reacted and reacted cells (*p* ≤ 0.001; Figure 1C). Specifically, in non-reacted spermatozoa, IZUMO1 was primarily distributed throughout the pre-equatorial subdomain and postacrosomal domain, with or without labeling in the apical ridge (P1 48.96%, P2 43.69%, respectively). In contrast, cells that had undergone the acrosome reaction exhibited IZUMO1 only in the postacrosomal domain (P3 43.10%) or was no longer detectable (P4 52.67%). No staining was observed when spermatozoa were incubated without the primary antibody (negative control; Figure 1A).

### 3.4 Quantitative analysis of IZUMO1 protein

**FIGURE 3:**
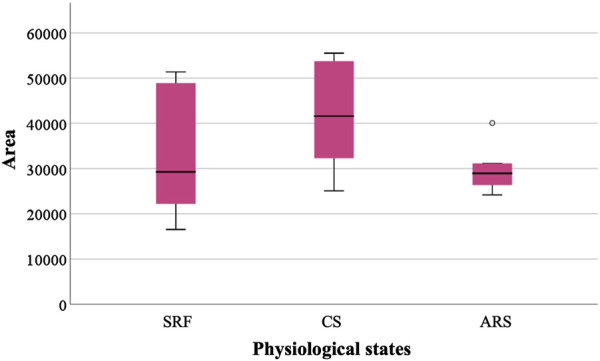
Relative abundance of IZUMO1 in three different physiological states based on the median peak area. Sperm-rich fraction (SRF), 1-h capacitated sperm selected by *swim-up* (CS), induced acrosome reaction in 1-h capacitated sperm (ARS). Circles in the graph indicate outlier values.

For the quantification of the protein of interest, DIA-NN filtered reports were used. Abundance values correspond to median peak area. IZUMO1 was present in all samples from different physiological states (Figure 3). SRF obtained an area of 32,921.25 while in CS the area increased to 41,632.58. After inducing the acrosome reaction (ARS) the area was settled as 29,938.92. The high sensitivity of the technique revealed a slight tendency despite no significant differences were observed between the conditions.

## 4 Discussion

Over the last decade, there has been a growing interest in identifying predictive genetic and protein markers with the aim of improving reproductive efficiency. Among several suggested essential proteins, IZUMO1 is noteworthy for its role in the adhesion and fusion of gametes; two critical steps to achieve successful fertilization. As part of its function, IZUMO1 organizes a protein complex comprised of TMEM81 and SPACA6 (Deneke et al., 2024; Elofsson et al., 2024). A monomeric form of IZUMO1 binds to his receptor, JUNO, located within the oolemma. JUNO rearranges IZUMO1 resulting in its dimerization. During this process, JUNO undergoes a positional change, leaving pockets through which another oocyte receptor can attach (Inoue et al., 2015). Recent research has suggested that MAIA is the receptor that interacts with the IZUMO1/JUNO complex, causing fusion between the juxtaposed membranes (Vondrakova et al., 2022). Interesting, there are not direct interactions between MAIA and IZUMO1 or JUNO (Bianchi et al., 2024). Therefore, a detailed understanding of these mechanisms could enable the regulation or control of gamete interactions, thereby preventing polyspermy. To achieve this, it is first necessary to investigate the presence and migration patterns of the IZUMO1 protein throughout the fertilization process.

**FIGURE 4:**
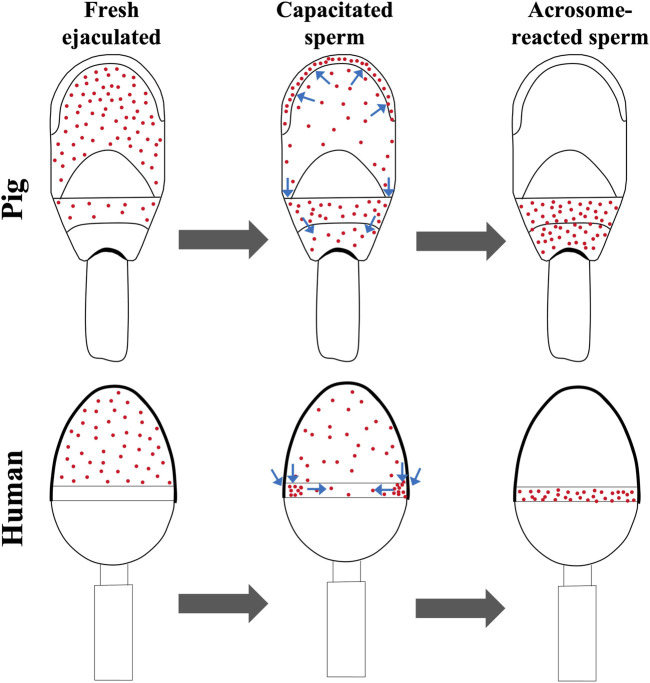
Working model for IZUMO1 diffusion in capacitated and acrosome reacted spermatozoa from pig and human. Pig IZUMO1 migrates from pre-equatorial subdomain to the apical ridge and postacrosomal domain. In contrast, due to the morphology, human IZUMO1 migrates from acrosomal region to the equatorial segment. Human data are based on our previous article (Gómez-Torres et al., 2023).

In the female reproductive tract, sperm selection occurs and only a few sperm will complete the capacitation process (Austin, 1974). Here, the capacitation was performed *in vitro* by *swim-up* selection (CS) whose adequacy was confirmed by tyrosine phosphorylation assay, obtaining an increase in the “high capacitated” and flagellum phosphorylation patterns (see Supplementary Datasheet S1). This process had a notable reduction of IZUMO1 protein clusters in the pre-equatorial subdomain, with an increase of a sperm subpopulation highlighted by the presence of IZUMO1 in the apical ridge (Figure 1); a subdomain generated by the folding of membranes to increase the contact surface between the spermatozoa and the ZP. Moreover, apical ridge is the site of the beginning of the acrosome reaction (Flesch and Gadella, 2000). The similarity of pattern distribution between SRF and CS agrees with the results obtained in our previous article about human spermatozoa (Gómez-Torres et al., 2023). Precisely, human IZUMO1 was mostly present in the acrosome of fresh ejaculated and capacitated cells. However, when we performed a sperm selection assay using hyaluronic acid, in matured spermatozoa IZUMO1 was observed in a speckled form in the acrosome with a labelled equatorial segment, reflecting the migration of IZUMO1. Similarly, the patterns found in boars (P1 and P2) are consecutive stages prior to the acrosomal reaction. Due to morphological differences between boar and human spermatozoa, IZUMO1 diffuse differently, as shown in Figure 4. In boars, the pre-equatorial subdomain is connected with the postacrosomal domain whereas the equatorial subdomain is a structure that is not exposed to the edges of the sperm head (Supplementary Datasheet S1). In contrast, the equatorial segment of human spermatozoa is a ring situated on the posterior edge of the acrosome which remains exhibited to the cell surface. In fact, human equatorial segment divides perfectly the acrosomal region and postacrosomal region (Zhu, 2020). Consequently, IZUMO1 in boars’ sperm migrates along the sides of the pre-equatorial subdomain to the postacrosomal domain while in humans it migrates to the equatorial segment. As can be seen in Supplementary Datasheet S1 the presence of IZUMO1 in the postacrosomal domain was corroborated performing a co-staining with an indicator of tyrosine phosphorylation, that never showed fluorescence in this domain (Jones et al., 2008).

The presence of IZUMO1 in porcine reacted spermatozoa was demonstrated a decade ago by analysis of subcellular fractions via Western blot (Kim et al., 2013). In addition, Tanihara et al. (2014) reported that spermatozoa that undergo spontaneously acrosome reaction had a lower penetration capacity than those that lost the acrosome by passing or that had passed the ZP. Here, we revealed that this process, simulated *in vitro* by calcium ionophore A23187 and calcium chloride (ARS1), promoted the localization of IZUMO1 to the postacrosomal domain (P3) or was lost (P4) (Figure 1). In accordance with these results, IZUMO1 has been observed in the postacrosomal region of mouse spermatozoa (Sebkova et al., 2014), despite others have localized it in the equatorial segment (Satouh et al., 2012). In fact, some authors have reported fusion at the postacrosomal region in hamster sperm cells (Yanagimachi and Noda, 1970a; Yanagimachi and Noda, 1970b), supporting the presence of IZUMO1 in this subdomain after acrosomal reaction in porcine.

Moreover, our immunolocalization results were supported by IZUMO1 proteomic analysis between different physiological states via liquid chromatography and tandem mass spectrometry (LC–MS/MS). Specifically, a slight increase in IZUMO1 protein in CS was observed, probably indicating a proper sperm selection. Furthermore, after inducing acrosome reaction there was a reduction in IZUMO1 relative abundance (CS: 41,632.58 vs. ARS: 29,938.92). When compared with fluorescence data, this reduction may be attributed to the relocalization of IZUMO1 in acrosome reacted spermatozoa.

## 5 Conclusion

IZUMO1 porcine protein locates in the pre-equatorial subdomain of noncapacitated sperm migrates to the apical ridge and the postacrosomal domain during early steps of fertilization process. Our proposed working model illustrates the differences between IZUMO1 diffusion depending on the sperm head morphology regions. Moreover, the presence of IZUMO1 in the postacrosomal domain of reacted sperm implies the functional role of this region during porcine gamete fusion.

## Data Availability

The original contributions presented in the study are included in the article/Supplementary Material, further inquiries can be directed to the corresponding author.
