# Supplementary material for: Immunolocalization and proteomic analyses of IZUMO1 in porcine spermatozoa
Source: Front Cell Dev Biol. 2025 May 16;13:1576881. doi: 10.3389/fcell.2025.1576881 (PMC12134388; doi:10.3389/fcell.2025.1576881)
Supplement: Supplementary file 1 [file DataSheet1.pdf]

Supplementary Material

Table S1. Statistical data of IZUMO1 distribution in SRF, CS, ARS. Data areexpressed as mean ± SD (standard deviation).

Pattern	SRF	CS	ARS
P1	49.31 ± 11.05	24.58 ± 15.45	21.47 ± 20.72
P2	44.32 ± 14.33	54.53 ± 13.20	2.74 ± 3.76
P3	1.21 ± 2.23	13.55 ± 11.35	51.55 ± 30.81
P4	5.16 ± 4.94	7.24 ± 6.08	24.25 ± 13.12

Pattern 1 (P1) characterized by speckled staining in the pre-equatorial subdomain and postacrosomal domain, pattern 2 (P2) displaying strong apical ridge staining with speckled staining in the pre-equatorial subdomain and postacrosomal domain, pattern 3 (P3) exhibiting speckled staining in the postacrosomal domain, and pattern 4 (P4) without labelling. Sperm-rich fraction (SRF), one-hour capacitated sperm selected by *swim-up* (CS), induced acrosome reaction in one-hour capacitated sperm (ARS).

Table S2. Statistical differences of IZUMO1 staining patterns between different sperm physiological conditions (SRF, CS, ARS).

Significance between Physiological Conditions			Pattern	Physiological Condition
SRF	CS	ARS		
-	***	***	P1	SRF
-	n.s.	***	P2	
-	n.s.	***	P3	
-	n.s.	***	P4	
	-	n.s.	P1	CS
	-	***	P2	
	-	***	P3	
	-	***	P4	
		-	P1	ARS
		-	P2	
		-	P3	
		-	P4	

Pattern 1 (P1) characterized by speckled staining in the pre-equatorial subdomain and postacrosomal domain, pattern 2 (P2) displaying strong apical ridge staining with speckled staining in the pre-equatorial subdomain and postacrosomal domain, pattern 3 (P3) exhibiting speckled staining in the postacrosomal domain, and pattern 4 (P4) without labelling. Sperm-rich fraction (SRF), one-hour capacitated sperm selected by *swim-up* (CS), induced acrosome reaction in one-hour capacitated sperm (ARS).

*** Bonferroni post hoc test p-value ≤ 0.001; n.s. not significant.

Table S3. Statistical data of tyrosine phosphorylation distribution in SRF and CS. Data are expressed as mean ± SD (standard deviation).

Pattern	SRF	CS
PI	13.58 ± 15.15	31.69 ± 10.81
PII	86.10 ± 14.84	48.02 ± 10.51
PIII	0.32 ± 0.56	20.29 ± 10.59
PIV	5.68 ± 3.50	69.35 ± 12.19

Pattern classification according to Luño *et al*. (2013). Sperm-rich fraction (SRF), one-hour capacitated sperm selected by *swim-up* (CS).

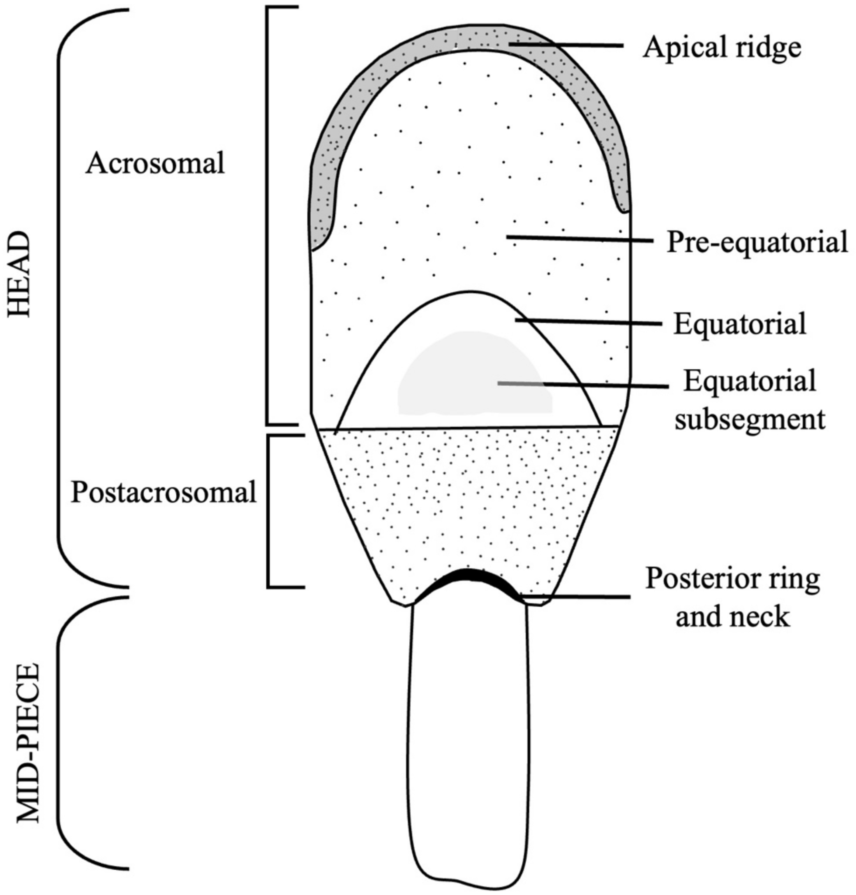

Figure S1. A surface view of the boar sperm head and mid-piece with the subdomains.

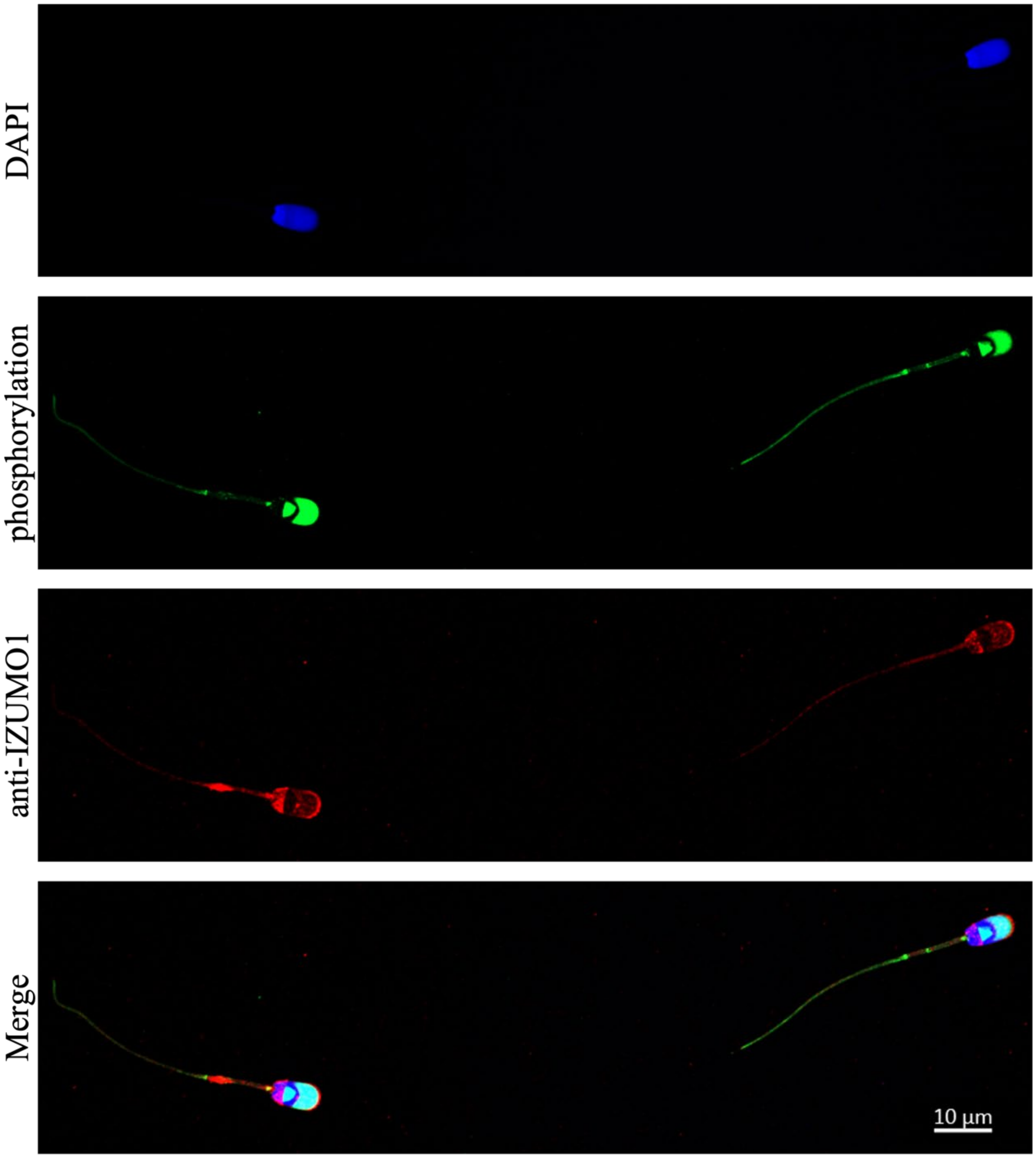

Figure S2. Co-staining of IZUMO1 and tyrosine phosphorylation using anti-4G10 antibody.
